# Meta-analyses of the association of *G6PC2* allele variants with elevated fasting glucose and type 2 diabetes

**DOI:** 10.1371/journal.pone.0181232

**Published:** 2017-07-13

**Authors:** Yuanyuan Shi, Yuqian Li, Jinjin Wang, Chongjian Wang, Jingjing Fan, Jingzhi Zhao, Lei Yin, Xuejiao Liu, Dongdong Zhang, Linlin Li

**Affiliations:** 1 Department of Epidemiology and Biostatistics, College of Public Health, Zhengzhou University, Zhengzhou, Henan, PR China; 2 Department of Clinical Pharmacology, School of Pharmaceutical Science, Zhengzhou University, Zhengzhou, Henan, PR China; 3 Discipline of Public Health and Preventive Medicine, Center of Preventive Medicine Research and Assessment, Henan University of Traditional Chinese Medicine, Zhengzhou, Henan, PR China; 4 Department of Endocrinology, Military Hospital of Henan Province, Zhengzhou, Henan, PR China; Indiana University Bloomington, UNITED STATES

## Abstract

**Objective:**

To collectively evaluate the association of glucose-6-phosphatase catalytic unit 2 (*G6PC2*) allele variants with elevated fasting glucose (FG) and type 2 diabetes (T2D).

**Design:**

Meta-analysis

**Data sources:**

PubMed, Web of Knowledge and Embase databases.

**Study selection:**

Full text articles of studies that identified an association of *G6PC2* with T2D and elevated FG.

**Patient involvement:**

There was no T2D patient involvement in the analyses on the association of FG with *G6PC2*, there were T2D patients and non-diabetes patient involvement in the analyses on the association of T2D with *G6PC2*.

**Statistical analysis:**

Random-effects meta-analyses were used to calculate the pool effect sizes. I^2^ metric and H^2^ tests were used to calculate the heterogeneity. Begg's funnel plot and Egger’s linear regression test were done to assess publication bias.

**Results:**

Of the 423 studies identified, 21 were eligible and included. Data on three loci (rs560887, rs16856187 and rs573225) were available. The G allele at rs560887 in three ethnicities, the C allele at rs16856187 and the A allele at rs573225 all had a positive association with elevated FG. Per increment of G allele at rs560887 and A allele at rs573225 resulted in a FG 0.070 mmol/l and 0.075 mmol/l higher (ß (95% CI) = 0.070 (0.060, 0.079), *p* = 4.635e-50 and 0.075 (0.065, 0.085), *p* = 5.856e-48, respectively). With regard to the relationship of rs16856187 and FG, an increase of 0.152 (95% CI: 0.034–0.270; *p* = 0.011) and 0.317 (95% CI: 0.193–0.442, *p* = 6.046e-07) was found in the standardized mean difference (SMD) of FG for the AC and CC genotypes, respectively, when compared with the AA reference genotype. However, the G-allele of rs560887 in Caucasians under the additive model and the C-allele of rs16856187 under the allele and dominant models were associated with a decreased risk of T2D (OR (95% CI) = 0.964 (0.947, 0.981), *p* = 0.570e-4; OR (95% CI) = 0.892 (0.832, 0.956), *p* = 0.001; and OR (95% CI) = 0.923(0.892, 0.955), *p* = 5.301e-6, respectively).

**Conclusions:**

Our meta-analyses demonstrate that all three allele variants of *G6PC2* (rs560887, rs16856187 and rs573225) are associated with elevated FG, with two variants (rs560887 in the Caucasians subgroup and rs16856187 under the allele and dominant model) being associated with T2D as well. Further studies utilizing larger sample sizes and different ethnic populations are needed to extend and confirm these findings.

## Introduction

Fasting plasma glucose (FPG) levels are associated with a risk of type 2 diabetes (T2D) and cardiovascular disease [1]. There is strong evidence suggesting that hyperglycemia is a risk factor in a dose-dependent manner for both micro- and macro-vascular complications in both type 1 and type 2 diabetes [2].

Both genetic and environmental factors contribute to the pathophysiology of T2D [3, 4]. However, the contribution of genetic factors to T2D risk is not well understood. Global knockout of theglucose-6-phosphatase catalytic unit 2 (*G6PC2*) gene in mice led to a significant decrease in blood glucose [5]. Previous studies have showed that higher FPG levels within the normal glucose range constitute an independent risk factor for T2D [1, 6]. Considering the genetic risk that might result from *G6PC2* alleles, a number of studies have explored the association of *G6PC2* with fasting glucose (FG) and T2D in different ethnicities [7–12]. However, individual studies have yielded inconsistent or conflicting findings, possibly caused by limitations associated with an individual study, such as different genetic backgrounds and ethnicity, sample size and so on. Wang H et al [13] have previously performed a meta-analysis on the association of *G6PC2* rs560887 with T2D. To expand and evaluate more precisely the relationship between *G6PC2* and FG and T2D, we carried out meta-analyses of published studies.

## Methods

### Search strategy

We included all studies published prior to 4st April of 2017 that reported an association between *G6PC2* and T2D and FG. Eligible studies were found by searching the PubMed, Web of Knowledge and Embase databases for relevant reports. We used the gene name “*G6PC2”* as search term limited in all fields to retrieve association studies between genetic variants in *G6PC2* and FG or T2D. We also reviewed reference lists of the identified publications for additional relevant studies. A literature search was performed on these databases without restriction to regions or publication types. Two investigators (YY.S and YQ.L) independently searched the articles, and disagreements were resolved by discussion.

### Selection

The study inclusion criteria were as follows: (1) published in Chinese or English; (2) primary outcomes of T2D or FG were given; (3) either I or II, as follows: (I) provided the odds ratio (OR) with 95% confidence interval (CI) or adequate information about the genotype and allele to calculate the OR and 95% CI for the association of rs568007 and rs16856187 polymorphisms with T2D. (II) provided mean and standard deviation (SD) values of FG and sample size (n) in every genotype for rs16856187, and linear regression coefficients (ß) of per-effect allele from linear regression analysis for the association of rs560887 and rs573225 with FG or enough data to calculate them. Studies were excluded if any of the following factors were identified: (1) not an association study for T2D or FG [14–24]; (2) studied other single nucleotide polymorphisms (SNPs) [25–28]; (3) data were not fully available [29–32]; (4) not population-based studies [33–34]; (5) meta-analyses or systematic review [13, 35–40]; (6) duplicate studies [41–43]. For duplicate publications, the study with the most recent and complete information was included.

### Patient involvement

There was no T2D patient involvement in the analyses on the association of FG with *G6PC2*; There were T2D patients and non-diabetes patient involvement in the analyses on the association of T2D with *G6PC2*.

### Data extraction

Data were extracted and summarized independently by two of the authors. The adjudicating senior authors resolved any disagreement. If the data were unavailable, an attempt was made to contact the corresponding author to request missing data via E-mail. The following information was extracted: (1) study characteristics such as the first author, study name, year published, country; (2) subjects and methods characteristics including sex, mean age, sample size (n), Body Mass Index (BMI), genotyping method and blood samples measured for FG; (3) primary outcomes such as risk allele, risk allele frequency (RAF), OR with 95% CI and their adjustment factors, statistical methods and the *p* value of Hardy-Weinberg equilibrium (HWE) test in control group for data on T2D; Mean and SD of FG and sample size (n) in every genotype, and ß and their standard error (SE) or 95% CI, *p* value for linear regression and their adjustment factors as in previously published studies [44–46], and the *p* value of HWE for data on FG. All data were extracted independently by two investigators (YY.S and YQ.L), and discrepancies were resolved by discussion.

### Quality assessment

The strengthening report of genetic association studies (STREGA) quality score system was used to assess the qualities of all included studies [47]. The STREGA system includes twenty-two quality assessment items with scores ranging from 0 to 22 (S1 Supplement). Studies are classified into three levels based on their scores: low quality (0–12), moderate-high quality (13–17), and high quality (18–22). Two authors (XJ.L and DD.Z) independently assessed the quality of included studies. Discrepancies over quality scores were resolved by discussing with all authors and subsequent consensus.

### Statistical analysis

In our meta-analyses the included studies on rs560887 and rs573225 used an additive model to assess the genetic effect of *G6PC2* polymorphisms [48]. For the studies on rs16856187, an additive model (AA versus AC versus CC) was used for the association of FG, and an allele model (A versus C), dominant model and recessive model were used for the association of T2D. The ß value and SEs were used to identify the association of rs560887 and rs573225 [14, 32, 44] with FG. SE of the ß value was calculated by 95% CI or ß value and *p* value when SE was not extracted directly from the original literature [49, 50]. The SMD was used to analyze the association between rs16856187 and FG. ORs with 95% CIs were assessed to determine the relationship between T2D and rs560887 and rs16856187.

The aggregated results OR and 95% CI, ß and 95% CI and SMD were calculated using random-effects meta-analysis. A statistical test for heterogeneity was conducted using the I^2^ metric and H^2^ tests [51]. A I^2^ greater than 50% or H^2^ greater than 1 was suggestive of substantial between-study heterogeneity [52–53]. Sensitivity analyses were performed by omitting each study to identify possible study contributions to the heterogeneity. To evaluate the reliability and stability of our results, Begg's funnel plot and Egger’s linear regression test were done to assess publication bias [54, 55]. We divided the study populations into three ethnic subgroups, including Caucasians, Asians and African-Americans for the relationship between FG and rs560887, and two ethnic subgroups (Caucasians and Asians) for the relationship between T2D and rs560887. All analyses were performed using Stata 12.1 (Stata Corp, College Station, TX, USA).

The HWE for all the subjects of each study was evaluated using χ^2^ test. For the studies which didn’t include the distributions of genotypes but contained the information on the RAF in both cases and controls, we calculated the frequency of the different genotypes according to the HWE Law, which can be used to calculate the crude ORs and 95% CIs under an additive genetic model. All reported probabilities (*p* values) were two-sided, with *p* < 0.05 considered statistically significant.

Finally, for a better presentation of the public health relevance, we explored the PAR by taking into account both the pooled per-allele ORs and the pooled RAF (T2D risk allele frequency). PAR was calculated as PAR = (X − 1)/X. Assuming a multiplicative model, X = (1 − f)^2^ + 2f(1 − f)γ + f^2^γ^2^, where f is RAF and γ is their estimated ORs. We calculated the pooled prevalence of each risk allele in various groups using the inverse variance method described previously [56].

### Power calculations

Power to detect a genetic association was estimated using the QUANTO program version 1.2.4. For the association study with FG, we had an estimated power of more than 99.99% to detect a minimal per-allele effect at β of 0.070 mmol/l for rs560887 and 0.075 mmol/l for rs573225 under an additive model, depending on an allele frequency of 0.76 and 0.67. For the association study with T2D, we had an estimated power of 97.58% to detect an OR of 0.967 for rs560887 under the prevalence of 8.8% [57] under an additive model, and 98.78%, 64.23% and 10.01% power to detect genetic effects at an OR of 0.960, 0.892 and 0.923 for rs16856187 under allele, dominant and recessive model, respectively. A *p* value <0.05 was considered statistically significant (two-tailed).

## Results

### Literature search results

**Fig 1:**
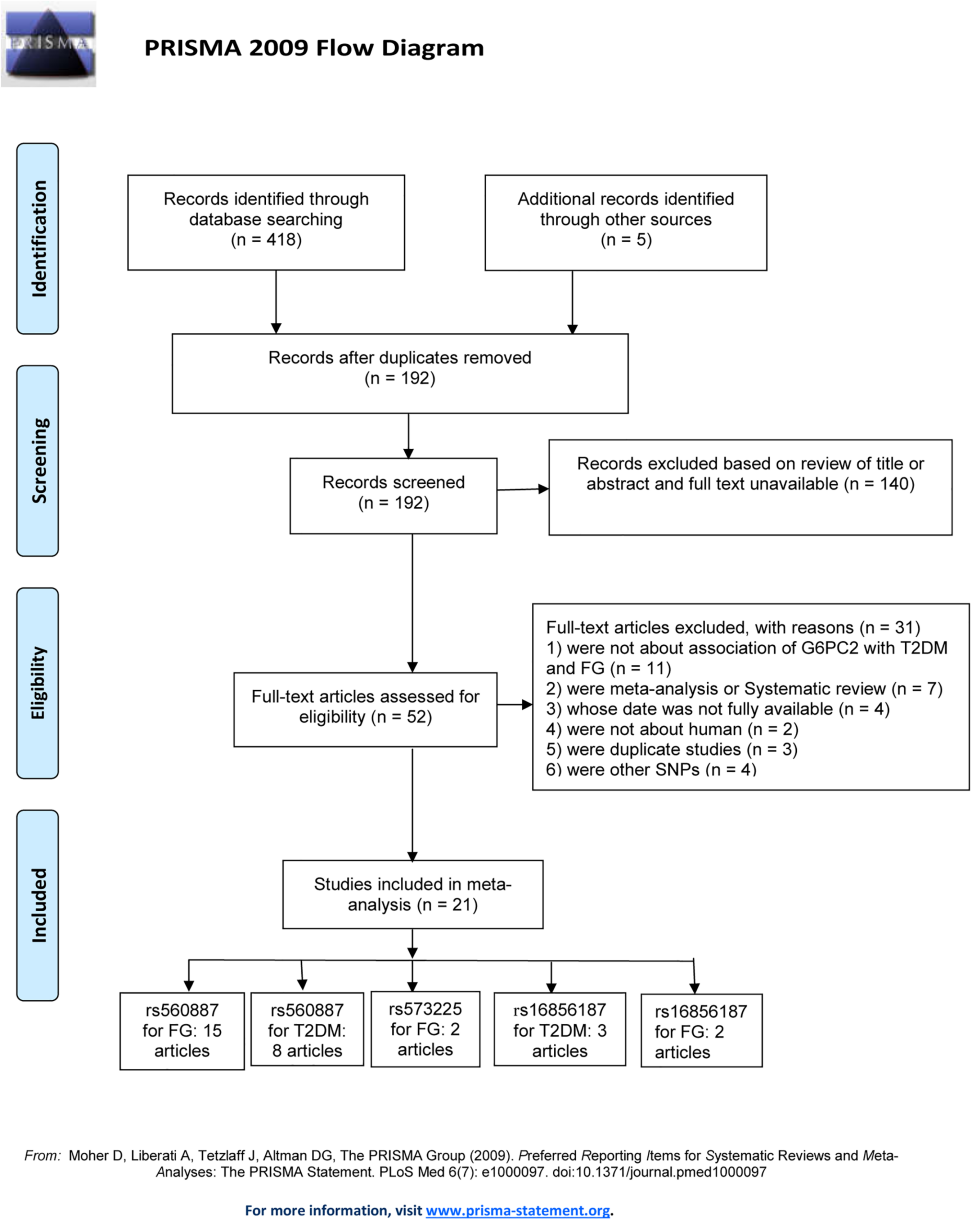
PRISMA 2009 flow diagram.

Through literature searches a total of 423 articles from PubMed (National Center for Biotechnology Information), Web of Knowledge and Embase databases were identified up to 4st Aprilof 2017. A flow chart of study selection in the meta-analyses is shown in Fig 1. There were 52 articles included after duplicates were removed and following the screening of the titles and abstracts. As shown in S2 Supplement, 31 full-text articles were excluded. Overall, 21 articles were eligible and included (see study inclusion flowchart in Fig 1[7–12, 58–72]). Of these, 5 articles covered the loci rs560887 for FG and T2D, 3 articles covered the loci rs16856187 for FG and T2D, 2 articles covered the loci rs560887 and rs573225 for FG, 8 articles covered the loci rs560887 for FG, and 3 articles covered the loci rs560887 for T2D, respectively (Tables 1 and 2). In total, 18 studies comprising 69120 cases (62492 for rs560887, 6628 for rs16856187), 126483 non-diabetic controls (119627 for rs560887, 6856 for rs16856187), and 35 studies containing 187968 non-diabetic participants (13752 for rs573225 and rs560887, 170772 for rs560887 and 3444 for rs16856187) were included. The meta-analyses were carried out according to the “Meta-analysis on Genetic Association Studies” statement (S3 Supplement).

**Table 1:** Characteristics of the studies on the association of two SNPs with T2D.

First Author	Study name	Country (Ethnicity)	Year published	FG-raising allele (frequencies)	Case			Control			STREGA score	HWE	rs560887	rs16856187
					N	Male%	Age	N	Male%	Age				
Bouatia-Naji *et al*.	European	France (Caucasian)	2008	G (0.70)	2792	62.2	50.4	4073	47.1	46.8	14	Yes	√	
Reiling*et al*.	New Hoorn Study	The Netherlands (Caucasian)	2009	G (0.692)	2628	55	64	2041	46	53	17	Yes	√	
Rose*et al*.	Inter99	Denmark(Caucasian)	2009	G (0.689)	1963	61.6	60.5	4913	46.5	46.4	16	Yes	√	
Takeuchi *et al*.	Japanese	Japan (Asian)	2009	G (0.971)	5629	NA	NA	6406	NA	NA	17	Yes	√	
Takeuchi*et al*.	Sri Lankan	Sri Lanka (Asian)	2009	G (0.907)	599	NA	NA	515	NA	NA	17	Yes	√	
Prokopenko *et al*.	DGI	Finland, Sweden (Caucasian)	2009	G (NA)	1464	NA	NA	1467	NA	NA	14	NA	√	
Prokopenko*et al*.	KORA	Germany (Caucasian)	2009	G (NA)	433	NA	NA	1438	NA	NA	14	NA	√	
Prokopenko*et al*.	Rotterdam	The Netherlands (Caucasian)	2009	G (NA)	1178	NA	NA	4761	NA	NA	14	NA	√	
Prokopenko*et al*.	WTCCC T2D	UK (Caucasian)	2009	G (NA)	1924	NA	NA	2938	NA	NA	14	NA	√	
Prokopenko*et al*.	CCC	UK (Caucasian)	2009	G (NA)	512	NA	NA	499	NA	NA	14	Yes	√	
Prokopenko*et al*.	ADDITION/ELY	Europe(Caucasian)	2009	G (NA)	852	NA	NA	1593	NA	NA	14	Yes	√	
Dupuis*et al*.	MAGIC	Europe (Caucasian)	2010	G (0.70)	40655	NA	NA	87022	NA	NA	10	Yes	√	
Rees*et al*.	UKADS	South Asian (Asian)	2011	G (0.82)	857	45.3	56.9	417	52.0	54.9	15	Yes	√	
Rees*et al*.	DGP	South Asian (Asian)	2011	G (0.84)	821	52.4	54.6	1167	52.9	56.3	15	Yes	√	
Al-Daghri et al.	RIYADH COHORT	Saudi Arabia (Caucasian)	2017	G (0.81)	185	52.0	59.4	377	51.0	37.1	15	Yes	√	
Hu *et al*.	Shanghai	China (Asian)	2008	C (0.285)	1876	52.4	61.2	1800	41.3	57.4	16	Yes		√
Hu*et al*.	Shanghai	China (Asian)	2010	C (0.294)	3410	54.9	60.33	3412	40.0	50.1	15	Yes		√
Tam*et al*.	Hong Kong	China (Asian)	2010	C (0.298)	1342	40.5	44.5	1644	45.4	24.6	19	Yes		√

NA = not available; √ represents this SNP was studied; HWE = Hardy-Weinberg equilibrium; No represents *p* value of HWE less than 0.05, Yes represents *p* value of HWE more than 0.05; Europe represents the country from Europe.

**Table 2:** Characteristics of the studies on the association of three SNPs with fasting glucose.

First Author	Study name	country (Ethnicity)	Year published	n	Age (SD), year	Sex	BMI (SD), kg/m2	STREGA score	HWE	rs560887	rs573225	rs16856187
					male/female	male%	male/female			FG-raising allele (frequencies)	FG-raising allele (frequencies)	FG-raising allele (frequencies)
Reiling *et al*.	New Hoorn Study	The Netherlands (Caucasian)	2009	2225	53 (7)	46	NA	17	Yes	G (0.693)		
Prokopenko *et al*.	CoLaus	Switzerland (Caucasian)	2009	5000	52.46 (10.72)/53.84 (10.73)	46	26.36 (3.84)/24.94 (4.63)	14	Yes	G (0.72)		
Prokopenko *et al*.	Framingham	USA (Caucasian)	2009	6479	45.9 (11.5)/46.0 (11.6)	46.0	27.7 (4.2)/25.9 (5.5)	14	Yes	G (0.70)		
Prokopenko *et al*.	Rotterdam	The Netherlands (Caucasian)	2009	2058	63.8 (5.5)/64.2 (6.1)	43	25.9 (2.8)/26.3 (3.8)	14	Yes	G (0.69)		
Prokopenko *et al*.	Sardinia	Italy (Caucasian)	2009	4305	44.08 (18.10)/43.19 (17.3)	43.8	26.15 (4.11)/24.75 (5.03)	14	Yes	G (0.63)		
Takeuchi *et al*.	Japanese	Japan (Asian)	2009	4813	48.8 (12.3)	58.2	22.9 (3.2)	17	Yes	G (0.97)		
Takeuchi *et al*.	Sri Lankan	Sri Lankan (Asian)	2009	2319	51.8 (8.1)	45.9	23.9 (4.3)	17	No	G (0.91)		
Chambers *et al*.	Indian Asian	India (Asian)	2009	5089	53.9 (10.6)	85	26.8 (4.2)	13	Yes	G (0.85)		
Chambers *et al*.	European whites	Finland (Caucasian)	2009	4462	31	47.6	24.6 (4.2)	13	Yes	G (0.69)		
Bouatia-Naji *et al*.	Haguenau	France (Caucasian)	2010	1201	22.27 (3.95)	48.1	22.63 (4.15)	15	Yes	G (NA)	A (NA)	
Bouatia-Naji *et al*.	DESIR	France (Caucasian)	2010	3483	46.87 (9.99)	46.5	24.29 (3.57)	15	Yes	G (NA)	A (NA)	
Bouatia-Naji *et al*.	NFBC86	Finland (Caucasian)	2010	4372	16 (0)	48.9	21.27 (3.56)	15	Yes	G (NA)	A (NA)	
Bouatia-Naji *et al*.	Obese children	France (Caucasian)	2010	476	10.89 (3.14)	47.9	28.42 (6.13)	15	Yes	G (NA)	A (NA)	
Ramos *et al*.	HUFS	Africa (African-American)	2010	927	46.1 (12.6)/46.9 (13.5)	42	28.3 (6.9)/31.4 (8.7)	14	Yes	G (0.957)		
Renström *et al*.	GLACIER	Sweden (Caucasian)	2010	1630	52.3 (8.8)	39.8	25.9 (4.1)	15	Yes	G (0.71)		
Dupuis et al.	MAGIC	Europe (Caucasian)	2010	76558	NA	NA	NA	10	Yes	G (0.70)		
Barker *et al*.	FRENCH controls	France (Caucasian)	2011	634	11.9 (2.4)/11.9 (2.2)	48.9	17.5 (2.2)/17.7 (2.5)	14	Yes	G (0.70)		
Barker *et al*.	EYHS	Denmark, Estonia (Caucasian)	2011	1934	11.9 (2.9)/12.0 (2.9)	46.3	18.4 (2.8)/18.5 (3.1)	14	Yes	G (0.70)		
Barker *et al*.	FRENCH cases	France (Caucasian)	2011	581	11.2 (2.9)/10.8 (3.4)	45.1	29.9 (6.4)/29.4 (6.6)	14	Yes	G (0.70)		
Barker *et al*.	Raine	Australia (Caucasian)	2011	1045	14.1 (0.2)/14.1 (0.2)	52.2	21.2 (4.2)/21.9 (4.2)	14	Yes	G (0.70)		
Barker *et al*.	ALSPAC	UK (Caucasian)	2011	1736	15.4 (0.3)/15.4 (0.3)	51.0	20.9 (3.3)/21.7 (3.7)	14	Yes	G (0.70)		
Rees *et al*.	South Asians	Pakistan (Asian)	2011	1163	56.3 (10.8)	52.9	24.3 (5.0)	15	Yes	G (0.84)		
Torvik *et al*.	CAU	European-Americans (Caucasian)	2012	2349	62.5 (10.3)	46.8	27.5 (4.9)	16	Yes	G (0.72)		
Torvik *et al*.	CHN	China (Asian)	2012	664	61.7 (10.4)	48.6	23.8 (3.3)	16	Yes	G (0.97)		
Torvik *et al*.	AFA	Africa (African-American)	2012	1366	61.8 (10.2)	45.1	29.8 (5.6)	16	No	G (0.93)		
Torvik *et al*.	HIS	Hispania (Caucasian)	2012	1171	60.7 (10.3)	48.0	29.0 (4.8)	16	Yes	G (0.86)		
Baerenwald *et al*.	DESIR cohort	France (Caucasian)	2013	4220	NA	NA	NA	17	Yes	G (0.695)	A (0.670)	
Zheng *et al*.	Caucasians	Caucasus (Caucasians)	2015	336	13.9	38.7	NA	15	Yes	G (0.723)		
Zheng *et al*.	Hispanics	Hispania (Caucasian)	2015	205	12.6	45.6	NA	15	Yes	G (0.834)		
Zheng *et al*.	African-Americans	Africa (African-American)	2015	211	13.4	40.8	NA	15	Yes	G (0.934)		
Horikoshi et al.	1000G	Europe (Caucasian)	2015	40091	57.9	43.0	NA	10	Yes	G (0.69)		
Langlois *et al*.	Mexican children and adolescents	Mexico (mixed)	2016	1421	9.25 (2.07)	53.1	19.67(4.22)	18	Yes	G(0.913)		
Tam *et al*.	Healthy Adults	China (Asian)	2010	583	41.4 (10.5)	45.5	22.9 (3.3)	19	Yes			A (0.303)
Tam *et al*.	Healthy Adolescents	China (Asian)	2010	1061	15.4 (1.9)	45.3	19.9 (3.5)	19	Yes			A (0.299)
Hu *et al*.	Shanghai	China (Asian)	2008	1800	57.35 (12.35)	41.3	23.57 (3.25)	19	Yes			A (0.303)

NA = not available; √ represents this SNP was studied

HWE = Hardy-Weinberg equilibrium

No represents *p* value of HWE less than 0.05, Yes represents *p* value of HWE more than 0.05

Europe represents the country from Europe

mixed represents a multi-ethnic nation.

### Association of rs560887, rs573225 and rs16856187 polymorphisms with FG

**Fig 2:**
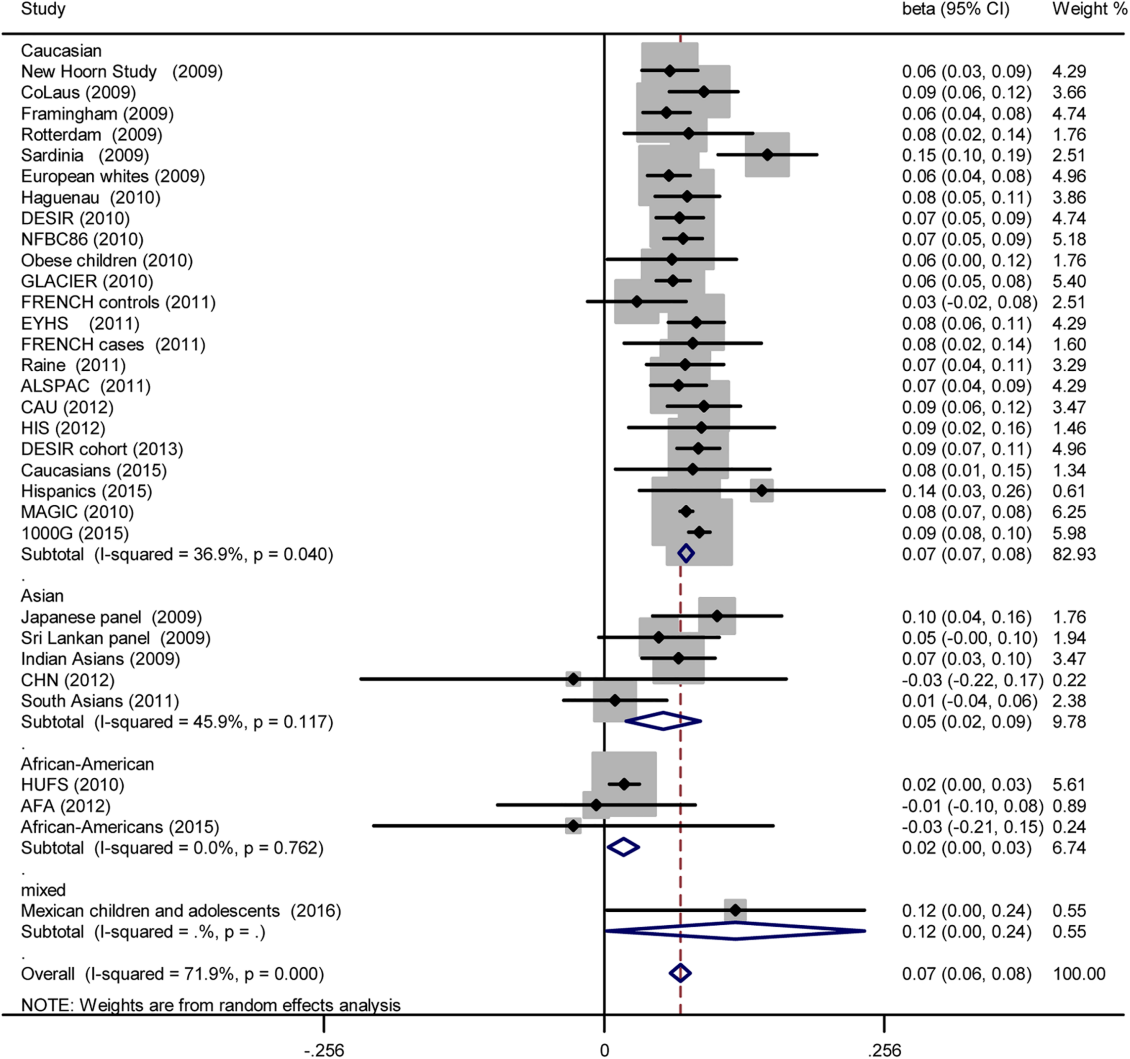
Forest plot for the association between rs560887 and FG under the additive model. Pooled ß for the additive genetic model are shown under a random-effects model. Square sizes were proportional to weight of each study in the meta-analyses. Significant association was detected in three ethnicities.

Meta-analysis estimates of SNP associations with FG are presented in Table 3. Under an additive model [48], a nominally significant positive association with FG was observed: per increment of additional G allele at rs560887 in *G6PC2*, FG was 0.070 mmol/l (95% CI: 0.060, 0.079, *p* = 4.635e-50) higher, with heterogeneity observed (I^2^ = 72%, 95% CI: 60%, 80%; H^2^ = 2.56). When the study population was divided into three ethnic subgroups, pooled ß (95% CI) and *p* were [0.075 (0.068, 0.081) mmol/l, *p* = 4.06e-118], [0.054 (0.020, 0.088) mmol/l, *p* = 0.002] and [0.018 (0.004, 0.031) mmol/l, *p* = 0.010] in Caucasians, Asians and African-Americans, respectively (Fig 2). Heterogeneity was observed [(I^2^ = 37%, 95% CI: 38%, 73%; H^2^ = 0.58), (I^2^ = 46%, 95% CI: 0, 80%; H^2^ = 0.85), (I^2^ = 0, 95% CI: 0, 90%; H^2^ = 0.00)] in Caucasians, Asians and African-Americans, respectively.

**Table 3:** Meta-analyses of *G6PC2* polymorphisms and FG or T2D.

	Association of *G6PC2* polymorphism with FG						Association of *G6PC2* polymorphism with T2D					
SNP/ FG-raising allele	number of studies	n	ß/SMD (95% CI)	*p*	I^2^% (95% CI)	*H*^*2*^	number of studies	n (case/control)	OR (95% CI)	*P*	I^2^% (95% CI)	H^2^
rs560887/G (overall)	32	184524	0.070 (0.060,0.079)	4.635e-50	72 (60,80)	2.56	15	24278/67043	0.967 (0.932,1.003)	0.076	39 (0,67)	0.42
Caucasians	23	78334	0.075 (0.068,0.081)	4.06e-118	37 (38,73)	0.58	11	16372/58538	0.964 (0.947,0.981)	0.570e-4	0 (0,60)	0.00
Asians	5	14048	0.054 (0.020,0.088)	0.002	46 (0,80)	0.85	4	7906/8505	1.120 (0.940,1.334)	0.205	66 (0,88)	2.06
African-Americans	3	2504	0.018 (0.004,0.031)	0.010	0 (0,90)	0.00						
Mexico	1	1421	0.120(0.002, 0.238)	0.046	**-**	**-**						
rs16856187/C	3	3444										
allele model#, (A vs C)							3	6628/6856	0.892 (0.832,0.956)	0.0013	35 (0,79)	0.50
Additive model#												
AC vs AA			0.152 (0.034,0.270) *	0.011	58 (0,88)	1.36						
CC vs AA			0.317 (0.193,0.442) *	6.046e-07	0 (0,90)	0.00						
Dominant model, (AC+CC) vs AA								6628/6856	0.923(0.892, 0.955)	5.301e-6	0 (0, 90)	0.00
Recessive model, CC vs (AC+AA)								6628/6856	0.960 (0.827,1.115)	0.596	80 (35, 94)	3.28
rs573225/A	5	13752	0.075 (0.065,0.085)	5.856e-48	0 (0,79)	0.00						

* indicate standardized mean differences (SMD) of AC vs AA and CC vs AA genotypes in rs16856187, respectively.

# indicate additive model for FG, allele model for T2D, respectively.

*p*: significance test of effect size (ß) = 0 or effect size (OR) = 1.

FG = fasting glucose; T2D = type 2 diabetic.

ß represents linear regression coefficients for the association of *G6PC2* polymorphism with FG.

OR represents odds ratio for the association of *G6PC2* polymorphism with T2D

ORs for rs560887 were calculated with logistic regression adjusted for different adjustment factors

ORs for rs16856187 were calculated using χ^2^ tests.

**Fig 3:**
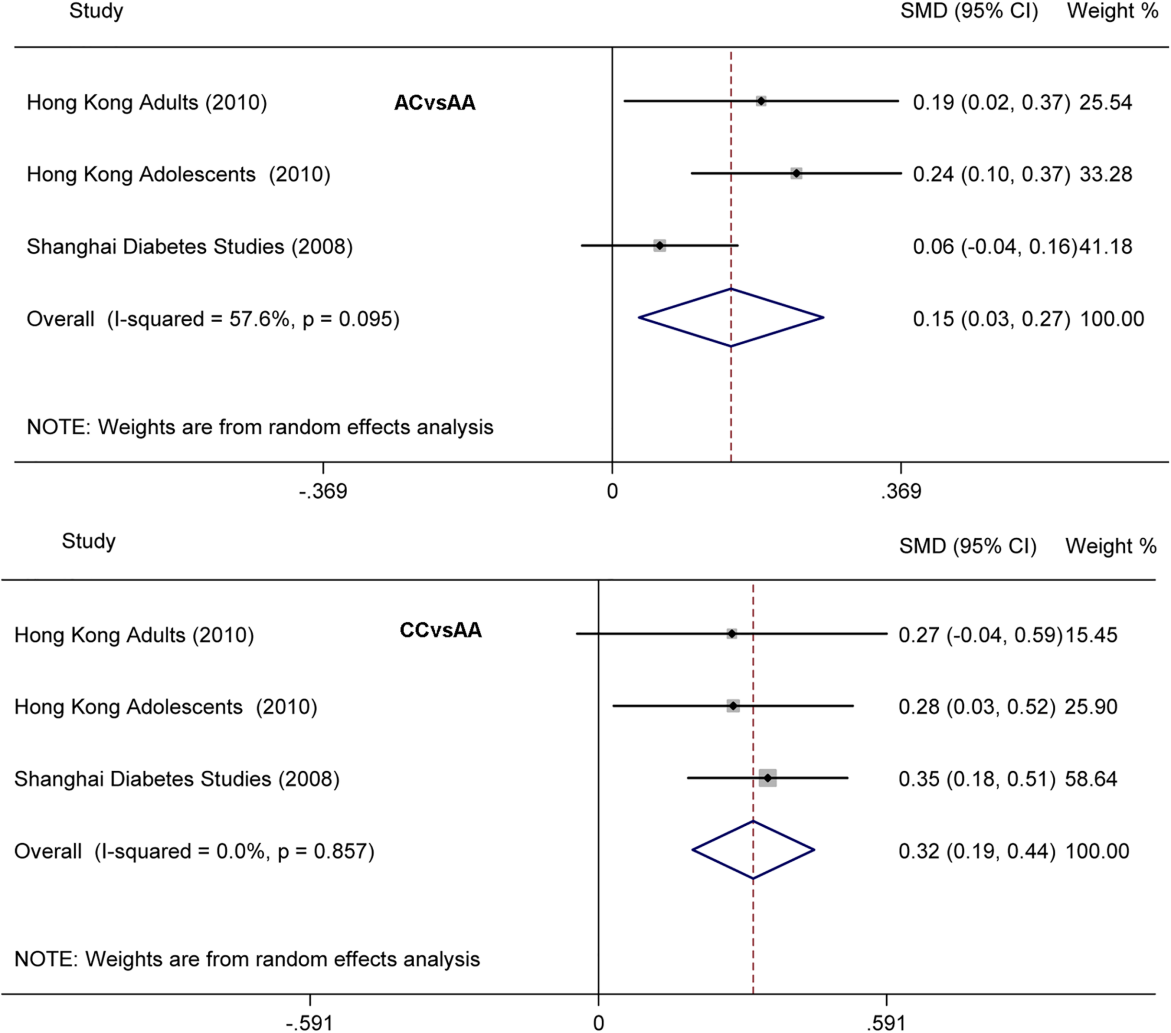
Forest plot for the association of rs16856187 with FG under the additive model.

In the association of rs16856187 with FG under the additive model, an additive trend of 0.152 (0.034, 0.270) and 0.317 (0.193, 0.442) increased in SMD of FG for AC and CC genotypes was found when compared to the AA reference genotype, respectively. Heterogeneity was observed (I^2^ = 58%, 95% CI: 0, 88%; H^2^ = 1.36) for AC vs AA while no heterogeneity was observed (I^2^ = 0, 95% CI: 0, 90%; H^2^ = 0.00) for CC vs AA (Fig 3).

**Fig 4:**
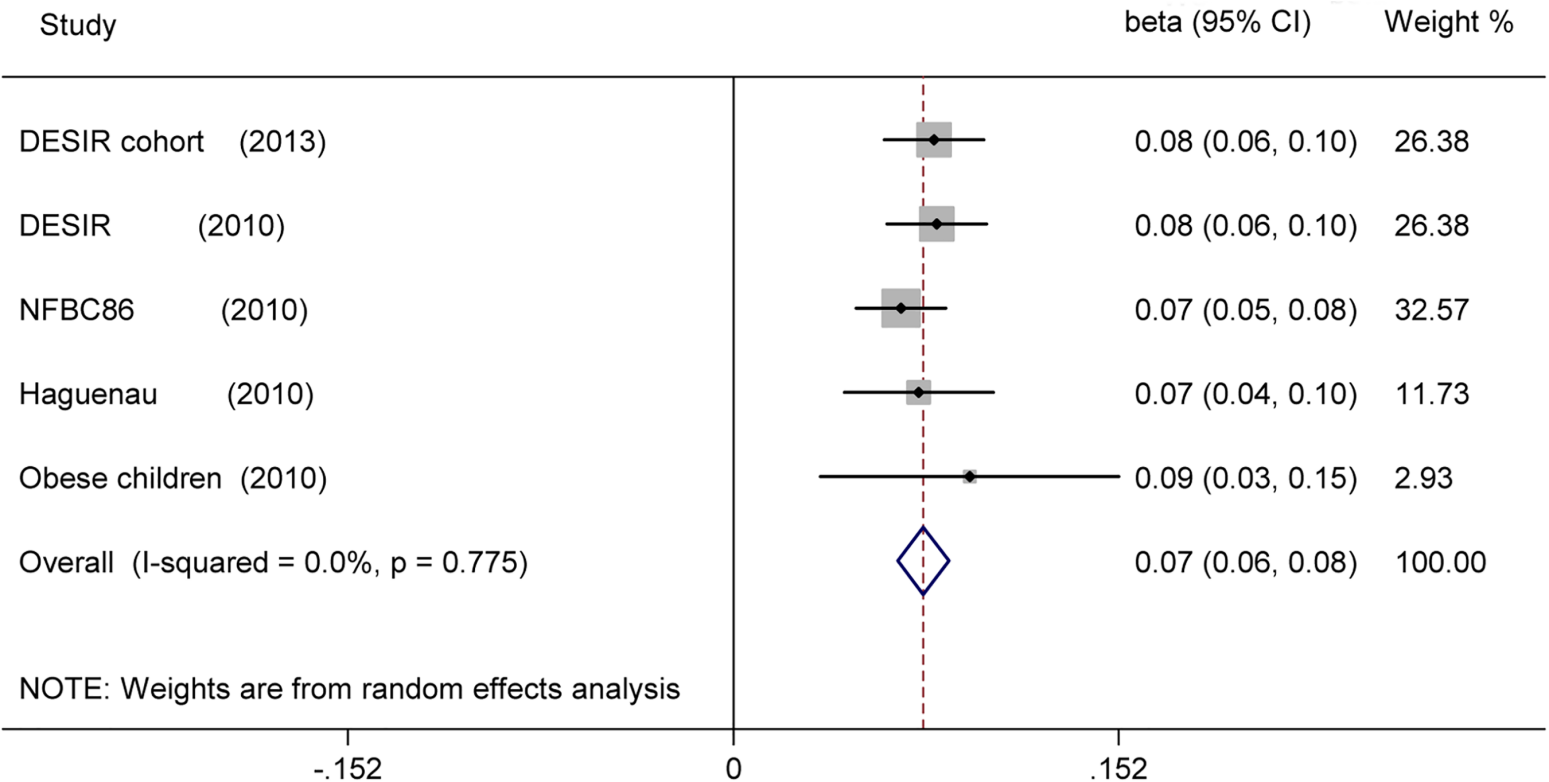
Forest plot for the association of rs573225 with FG under the additive model. Pooled ß for the additive genetic model was shown under a random-effects model. Square sizes were proportional to weight of each study in the meta-analysis.

In the association of rs573225 with FG, a nominally significant positive association with elevated FG was observed: per increment of A allele at rs573225 in *G6PC2*, FG was 0.075 mmol/L higher (ß = 0.075; 95% CI: 0.065–0.085, *p* = 5.856e-48), with no heterogeneity observed (I^2^ = 0, 95% CI: 0, 79%; H^2^ = 0.00) (Fig 4).

### Association of rs568007 and rs16856187 polymorphisms with T2D

**Fig 5:**
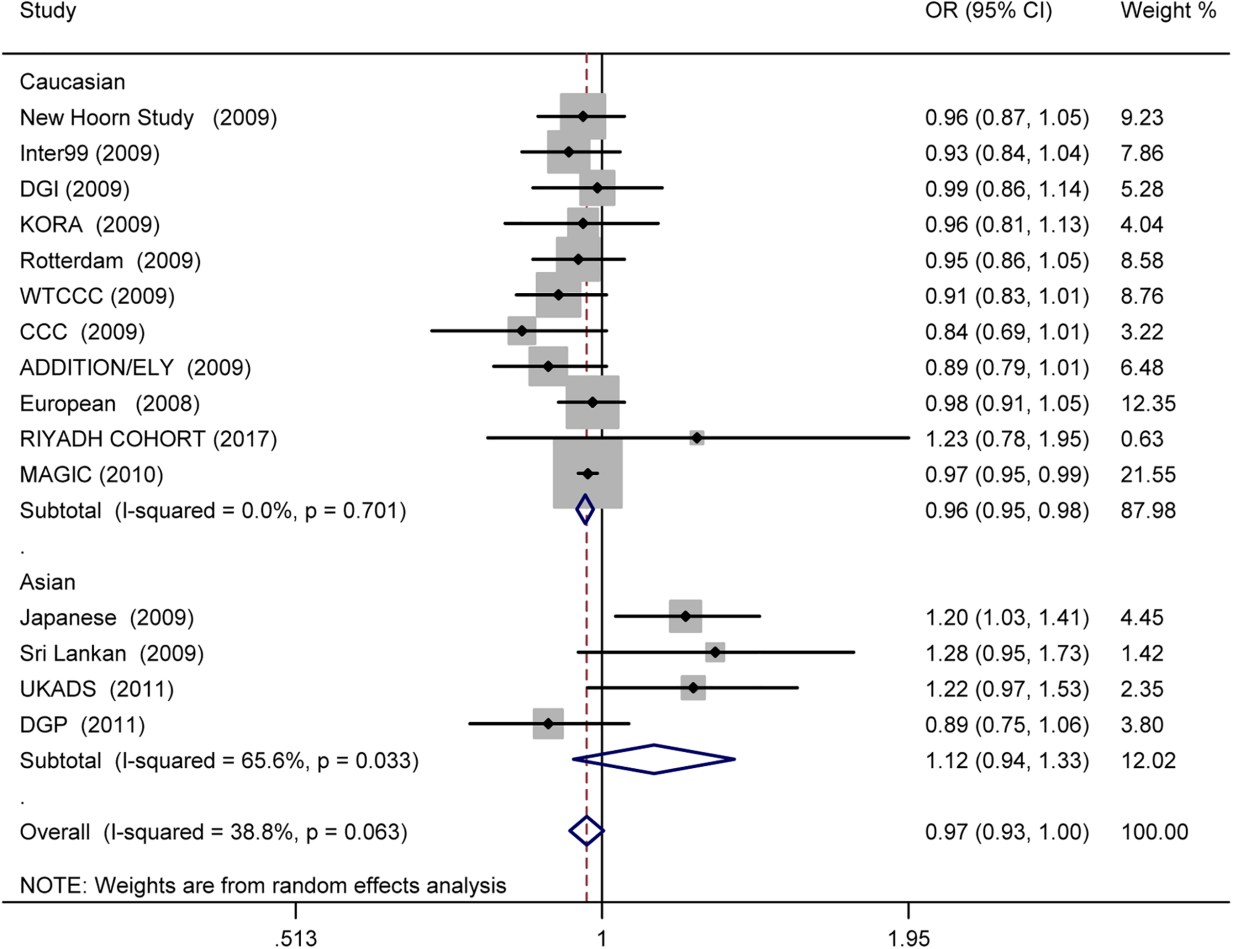
Forest plot for the association of *G6PC2* rs560887 with T2D under the additive model. Pooled OR for the additive genetic model was shown under a random-effects model, square sizes were proportional to the weight of each study in the meta-analysis.

In the overall estimate, no association was detected between the rs560887 and risk of T2D (OR = 0.967; 95% CI: 0.932–1.003; *p* = 0.076), with low heterogeneity (I^2^ = 39%, 95% CI: 0, 67%; H^2^ = 0.42). In Asians, rs560887 also had no association with risk of T2D (OR = 1.120; 95%CI: 0.940–1.334; *p* = 0.205) (Fig 5). Conversely, in the Caucasian subgroup we found a significant association between the FPG-raising G-allele and decreased risk of T2D (OR = 0.964; 95% CI: 0.947–0.981; *p* = 0.570e-4), with no heterogeneity observed (I^2^ = 0, 95% CI: 0, 60%; H^2^ = 0.00). When both the pooled RAF and the pooled per-allele OR were taken into account, the presence of each risk allele would be associated with a 5.4%, 5.3% and 18.3% increase in incidence of T2D according to the PAR estimate in total sample, Caucasian and Asians subgroup, respectively.

**Fig 6:**
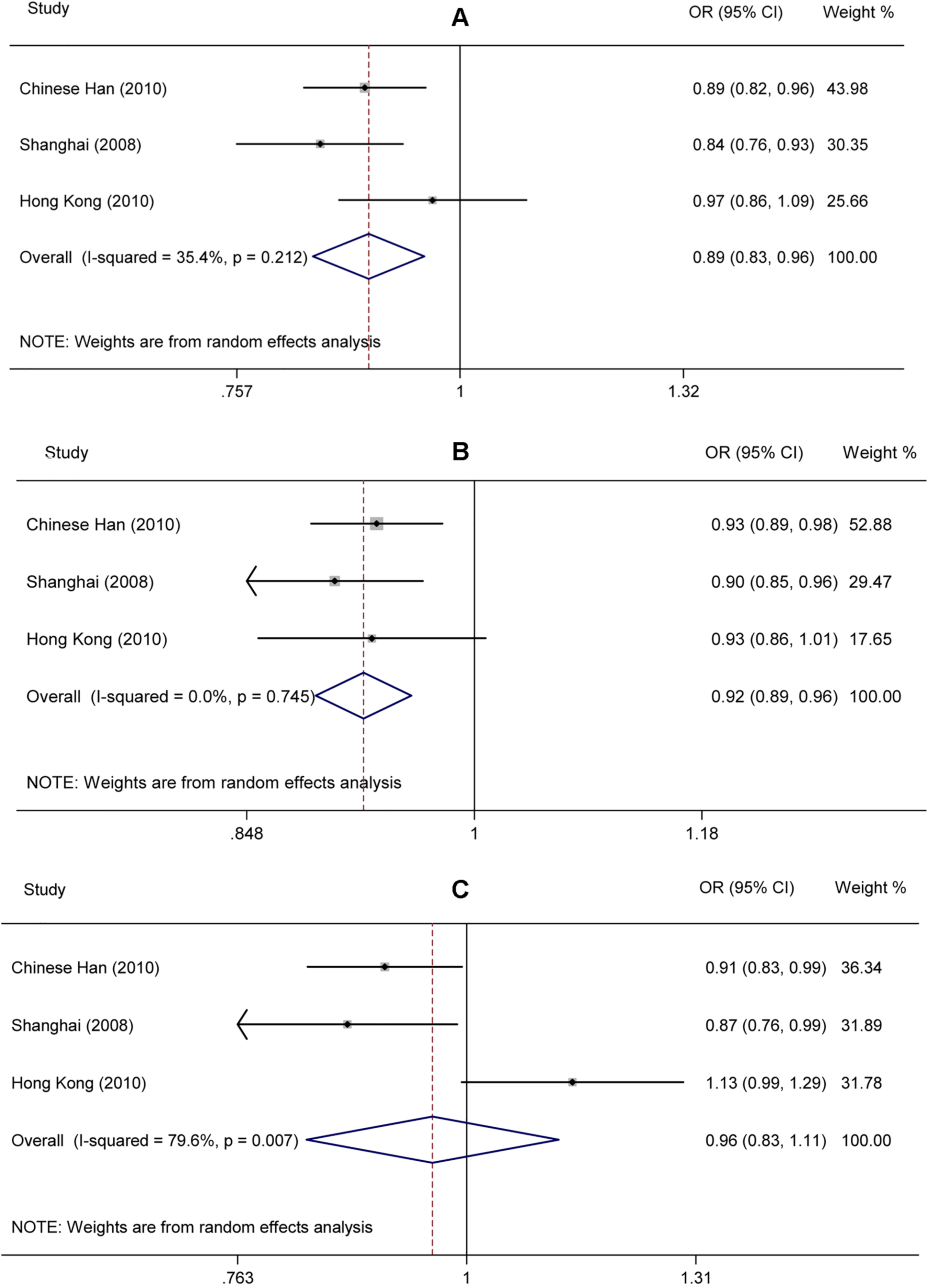
Forest plot for the association between *G6PC2* rs16856187 and T2D under the allele (A), dominant (B) and recessive (C) model.

Under the allele model, the association between the rs16856187-C allele and decreased risk of T2D was significant (OR = 0.892; 95% CI: 0.832–0.956; *p* = 0.001) with low heterogeneity among studies (I^2^ = 35, 95% CI: 0, 79%; H^2^ = 0.50) (Fig 6A). Under the dominant model (AC+CC vs AA), a significant negative association was detected (OR = 0.923; 95% CI: 0.892–0.955; *p* = 5.301e-6) with no heterogeneity among studies (I^2^ = 0, 95% CI: 0, 90%; H^2^ = 0.00) (Fig 6B). Under the recessive model (CC vs AC+AA), no significant association was detected (OR = 0.960; 95% CI: 0.827, 1.115; *p* = 0.596) with high heterogeneity among studies (I^2^ = 80, 95% CI: 35%, 94%; H^2^ = 3.28) (Fig 6C). Results under the allele and dominant model indicated that the FPG-raising C-allele might be associated with a decreased risk of T2D. When both the pooled RAF (rs16856187-A allele) and the pooled per-allele OR were taken into account, the presence of each A-allele would be associated with a 6.5%, 4.6% and 2.3% increase in incidence of T2D according to the PAR estimate under allele, dominant and recessive model, respectively.

### Publication bias, sensitivity test

Visual inspection of funnel plots for the primary outcomes did not show distinct asymmetry, and based on Begg’s funnel plots (S1 Fig) and Egger’s linear regression, no publication bias was observed (all *p* > 0.1). In the sensitivity test (S2 Fig), the leave-one-out influential analyses did not show any major change in the primary outcome, indicative of a good stability of results.

## Discussion

To the best of our knowledge, this is the most comprehensive meta-analyses on the evaluation of the associations between *G6PC2* SNPs and FG and T2D. Our meta-analyses include the most SNPs in *G6PC2* on the associations of these SNPs with FG and T2D studied to date. In these meta-analyses, we analyzed three SNPs (rs560887, rs16856187 and rs573225) in the *G6PC2* gene for an effect on FG and two SNPs (rs560887 and rs16856187) for an association with T2D. We found that all three SNPs were associated with elevated FG level in participants with normal glucose regulation, and rs560887 in the Caucasians subgroup and rs16856187 under allele and dominant model were all associated with T2D.

However, no associations with T2D risk were found for G at rs560887 in overall and Asians populations, which is consistent with a meta-analysis published in 2013[13]. Compared with this previous study, our study contained greater sample sizes in the Caucasians subgroup (number of case/control were 47673/97909 and 54586/111122 for previous and current studies, respectively). Association of G at rs560887 with T2D and FG in Caucasians is similar to individuals of European descent in the MAGIC study [OR (95% CI): 0.97 (0.95–0.99), ß (SE):0.075 (0.003) mmol/l, respectively] [70].

There may be some reasonable explanations for these differences between ethnic groups. First, the gene-gene interactions and different environmental factors may affect susceptibility to the genetic variant and diabetes [73, 74]. Second, the sample size for Asian populations may be too small. Third, a previous study has reported that Asian subgroups have unique risk-factor profiles for developing diabetes, which differ from other populations [75]. Thus, further investigations on Asian populations are needed to replicate the observed association with type 2 diabetes. We will explore the association of *G6PC2* with T2D in the Chinese population in the near future.

*G6PC2* belongs to the *G6PC* family of proteins, which catalyze the dephosphorylation of glucose-6-phosphate to glucose [76]. Thus, glucose-6-phosphatase activity could control glucose metabolism and insulin secretion [76]. However, carriers of the G allele at rs560887 in Caucasians subgroup and A allele at rs16856187 in allele and dominant model all displayed a lower risk of type 2 diabetes and a higher risk of elevated FPG level, which is inconsistent with these previous studies [1, 6]. The mechanism linking the SNP rs560887-A to reduced *G6PC2* activity might be connected to the relative expression of the full-length active protein [23, 77]. Studies have shown that heightened beta-cell sensitivity to glucose and a lowered glucose set-point for insulin secretion are early steps toward ß cell apoptosis [78]. Recent reports in individuals of European descent also demonstrated a strong association between *G6PC2* variants and insulin secretion [32]. The allele that decreased FPG was also found to lower beta cell function [65]. However, carriers of G at *G6PC2* rs560887 displayed a higher risk of type 2 diabetes and a higher FPG level in Asians. It is unclear whether ethnic differences in beta cell function [79, 80] contributed to the different results. Moreover, our relatively small sample size of Asians may also limit our ability to reach a reliable conclusion.

In addition, we also found that the presence of T at rs13387347, A at rs2232316, G at rs492594, A at rs483234, T at rs3755157 and C at rs478333 in *G6PC2* among Asians were correlated with a higher risk of T2D [10, 65, 69]. Meanwhile, C at rs478333 in adolescents, T at rs3755157 and A at rs483234 among Asians displayed a higher FG level. However, T at rs13387347 displayed a lower FG level [10, 65, 69]. Due to a lack of data, a meta-analysis was not completed for these SNPs, yet they still provide evidence for the association of *G6PC2* with FG and T2D.

This study shows that ß value (linear regression coefficient) rather than SMD for the association of FG with *G6PC2* SNPs (rs560887 and rs573225) when pooled, which was not seen in the previous studies. This is currently the most comprehensive meta-analyses on *G6PC2*.

Some limitations in our meta-analyses should be mentioned. First, our results on FG and T2D were based on slightly different adjusted estimates. Second, the studies included in the analyses may be insufficient to allow firm conclusions. Thus, potential publication bias is likely to exist, in spite of the lack of evidence for this obtained from our statistical tests. The power to detect bias is limited, particularly for moderate amounts of bias or meta-analyses based on a small number of small studies [81]. Third, heterogeneity is also a potential problem, with estimates of zero or even just low heterogeneity being a concern since heterogeneity is very likely present but undetected [82]. Finally, the sample size for rs16856187 is small, and the estimate of the effect of rs16856187 on FG may be imprecise. Therefore, further study is necessary to confirm this finding.

## Supporting information

S1 Supplement STREGA reporting recommendations, extended from STROBE statement. (DOC)

S2 Supplement Full-text articles excluded with reasons. (DOC)

S3 Supplement Meta-analysis on Genetic Association Studies checklist. (DOC)

S4 Supplement PRISMA 2009 checklist. (DOC)

S1 Fig Funnel plot of publication bias for the association of rs560887 (A), rs16856187 (CCvsAA) (B) and (ACvsAA) (C), rs573225 (D) with FG, rs560887 (E), rs16856187 under allele (F), dominant (G) and recessive (H) with T2D, respectively.
(TIF)

S2 Fig Sensitivity tests for the association of rs560887 (A), rs16856187 (CCvsAA) (B) and (ACvsAA) (C), rs573225 (D) with FG, rs560887 (E), rs16856187 under allele (F), dominant (G) and recessive (H) with T2D, respectively.
(TIF)
