# Supplementary material for: Meta-analyses of the association of G6PC2 allele variants with elevated fasting glucose and type 2 diabetes
Source: PLoS One. 2017 Jul 13;12(7):e0181232. doi: 10.1371/journal.pone.0181232 (PMC5509327; doi:10.1371/journal.pone.0181232)
Supplement: S2 Supplement — (DOC) [file pone.0181232.s002.doc]

**S2 Supplement List of Excluded Articles**

31 full-text articles were excluded, with reasons:

1) 11 articles were not about association of G6PC2 with T2DM and FG

2) 7 articles were meta-analysis or systematic review

3) 4 articles whose date were not fully available

4) 2 articles were not about human

5) 3 articles were duplicate studies

6) 4 articles were other SNPs

1. Benn M, Tybjaerg-Hansen A, McCarthy MI, Jensen GB, Grande P, Nordestgaard BG. Nonfasting glucose, ischemic heart disease, and myocardial infarction: a Mendelian randomization study. J Am Coll Cardiol. 2012;59(25):2356-65. Epub 2012/06/16. doi: 10.1016/j.jacc.2012.02.043. PubMed PMID: 22698489; PubMed Central PMCID: PMCPmc4606982.

2. Grimsby JL, Porneala BC, Vassy JL, Yang Q, Florez JC, Dupuis J, et al. Race-ethnic differences in the association of genetic loci with HbA1c levels and mortality in U.S. adults: the third National Health and Nutrition Examination Survey (NHANES III). BMC medical genetics. 2012;13:30. Epub 2012/05/01. doi: 10.1186/1471-2350-13-30. PubMed PMID: 22540250; PubMed Central PMCID: PMCPmc3433372.

3. Borowiec M, Fendler W, Dusatkova P, Antosik K, Pruhova S, Cinek O, et al. HbA1c-based diabetes diagnosis among patients with glucokinase mutation (GCK-MODY) is affected by a genetic variant of glucose-6-phosphatase (G6PC2). Diabetic medicine : a journal of the British Diabetic Association. 2012;29(11):1465-9. Epub 2012/04/11. doi: 10.1111/j.1464-5491.2012.03671.x. PubMed PMID: 22486180.

4. Huopio H, Cederberg H, Vangipurapu J, Hakkarainen H, Paakkonen M, Kuulasmaa T, et al. Association of risk variants for type 2 diabetes and hyperglycemia with gestational diabetes. Eur J Endocrinol. 2013;169(3):291-7. Epub 2013/06/14. doi: 10.1530/eje-13-0286. PubMed PMID: 23761423.

5. Pare G, Chasman DI, Parker AN, Nathan DM, Miletich JP, Zee RY, et al. Novel association of HK1 with glycated hemoglobin in a non-diabetic population: a genome-wide evaluation of 14,618 participants in the Women's Genome Health Study. PLoS Genet. 2008;4(12):e1000312. Epub 2008/12/20. doi: 10.1371/journal.pgen.1000312. PubMed PMID: 19096518; PubMed Central PMCID: PMCPmc2596965.

6. Rasmussen-Torvik LJ, Li M, Kao WH, Couper D, Boerwinkle E, Bielinski SJ, et al. Association of a fasting glucose genetic risk score with subclinical atherosclerosis: The Atherosclerosis Risk in Communities (ARIC) study. Diabetes. 2011;60(1):331-5. Epub 2010/11/03. doi: 10.2337/db10-0839. PubMed PMID: 21036910; PubMed Central PMCID: PMCPmc3012190.

7. Sanda S, Wei S, Rue T, Shilling H, Greenbaum C. A SNP in G6PC2 predicts insulin secretion in type 1 diabetes. Acta Diabetol. 2013;50(3):459-62. Epub 2012/03/23. doi: 10.1007/s00592-012-0389-y. PubMed PMID: 22438186.

8. Bonnefond A, Bouatia-Naji N, Simon A, Saint-Martin C, Dechaume A, de Lonlay P, et al. Mutations in G6PC2 do not contribute to monogenic forms of early infancy diabetes and beta cell dysfunction. Diabetologia. 2009;52(5):982-5. Epub 2009/02/25. doi: 10.1007/s00125-009-1299-6. PubMed PMID: 19238352.

9. Dos Santos C, Bougneres P, Fradin D. A single-nucleotide polymorphism in a methylatable Foxa2 binding site of the G6PC2 promoter is associated with insulin secretion in vivo and increased promoter activity in vitro. Diabetes. 2009;58(2):489-92. Epub 2008/11/06. doi: 10.2337/db08-0587. PubMed PMID: 18984742; PubMed Central PMCID: PMCPmc2628624.

10. Dogra RS, Vaidyanathan P, Prabakar KR, Marshall KE, Hutton JC, Pugliese A. Alternative splicing of G6PC2, the gene coding for the islet-specific glucose-6-phosphatase catalytic subunit-related protein (IGRP), results in differential expression in human thymus and spleen compared with pancreas. Diabetologia. 2006;49(5):953-7. Epub 2006/03/08. doi: 10.1007/s00125-006-0185-8. PubMed PMID: 16520917.

11. Chen P, Takeuchi F, Lee JY, Li H, Wu JY, Liang J, et al. Multiple nonglycemic genomic loci are newly associated with blood level of glycated hemoglobin in East Asians. Diabetes. 2014;63(7):2551-62. Epub 2014/03/22. doi: 10.2337/db13-1815. PubMed PMID: 24647736; PubMed Central PMCID: PMCPmc4284402.

12. Wessel J, Chu AY, Willems SM, Wang S, Yaghootkar H, Brody JA, et al. Low-frequency and rare exome chip variants associate with fasting glucose and type 2 diabetes susceptibility. Nature communications. 2015;6. doi: 10.1038/ncomms6897. PubMed PMID: WOS:000348741700001.

13. Mahajan A, Sim X, Ng HJ, Manning A, Rivas MA, Highland HM, et al. Identification and Functional Characterization of G6PC2 Coding Variants Influencing Glycemic Traits Define an Effector Transcript at the G6PC2-ABCB11 Locus. PLoS genetics. 2015;11(1). doi: 10.1371/journal.pgen.1004876. PubMed PMID: WOS:000349314600012.

14. Wang H, Liu L, Zhao J, Cui G, Chen C, Ding H, et al. Large scale meta-analyses of fasting plasma glucose raising variants in GCK, GCKR, MTNR1B and G6PC2 and their impacts on type 2 diabetes mellitus risk. PLoS One. 2013;8(6):e67665. Epub 2013/07/11. doi: 10.1371/journal.pone.0067665. PubMed PMID: 23840762; PubMed Central PMCID: PMCPmc3695948.

15. Marcolongo P, Fulceri R, Gamberucci A, Czegle I, Banhegyi G, Benedetti A. Multiple roles of glucose-6-phosphatases in pathophysiology: state of the art and future trends. Biochimica et biophysica acta. 2013;1830(3):2608-18. Epub 2012/12/26. doi: 10.1016/j.bbagen.2012.12.013. PubMed PMID: 23266497.

16. Adeva M, Gonzalez-Lucan M, Seco M, Donapetry C. Enzymes involved in l-lactate metabolism in humans. Mitochondrion. 2013;13(6):615-29. Epub 2013/09/14. doi: 10.1016/j.mito.2013.08.011. PubMed PMID: 24029012.

17. Edghill EL, McCulloch L, Fulton P, Beer N, Hattersley AT, Gloyn AL. Mutations in the third gene shown to alter fasting glucose levels in the population (G6PC2) are not a common cause of monogenic forms of pancreatic B-cell dysfunction. Diabetic medicine : a journal of the British Diabetic Association. 2009;26(1):113-4. Epub 2009/01/08. doi: 10.1111/j.1464-5491.2008.02618.x. PubMed PMID: 19125775.

18. van de Bunt M, Gloyn AL. From genetic association to molecular mechanism. Curr Diab Rep. 2010;10(6):452-66. Epub 2010/09/30. doi: 10.1007/s11892-010-0150-2. PubMed PMID: 20878272.

19. Florez JC, Jablonski KA, McAteer JB, Franks PW, Mason CC, Mather K, et al. Effects of genetic variants previously associated with fasting glucose and insulin in the Diabetes Prevention Program. PloS one. 2012;7(9):e44424. Epub 2012/09/18. doi: 10.1371/journal.pone.0044424. PubMed PMID: 22984506; PubMed Central PMCID: PMCPmc3439414.

20. Heni M, Ketterer C, Hart LM, Ranta F, van Haeften TW, Eekhoff EM, et al. The impact of genetic variation in the G6PC2 gene on insulin secretion depends on glycemia. The Journal of clinical endocrinology and metabolism. 2010;95(12):E479-84. Epub 2010/09/10. doi: 10.1210/jc.2010-0860. PubMed PMID: 20826583.

21. Demirci FY, Dressen AS, Hamman RF, Bunker CH, Kammerer CM, Kamboh MI. Association of a common G6PC2 variant with fasting plasma glucose levels in non-diabetic individuals. Annals of nutrition & metabolism. 2010;56(1):59-64. Epub 2009/12/24. doi: 10.1159/000268019. PubMed PMID: 20029179; PubMed Central PMCID: PMCPmc2855271.

22. Li X, Shu YH, Xiang AH, Trigo E, Kuusisto J, Hartiala J, et al. Additive effects of genetic variation in GCK and G6PC2 on insulin secretion and fasting glucose. Diabetes. 2009;58(12):2946-53. Epub 2009/09/11. doi: 10.2337/db09-0228. PubMed PMID: 19741163; PubMed Central PMCID: PMCPmc2780888.

23. Pound LD, Oeser JK, O'Brien TP, Wang Y, Faulman CJ, Dadi PK, et al. G6PC2: a negative regulator of basal glucose-stimulated insulin secretion. Diabetes. 2013;62(5):1547-56. Epub 2013/01/01. doi: 10.2337/db12-1067. PubMed PMID: 23274894; PubMed Central PMCID: PMCPmc3636628.

24. O'Brien RM. Moving on from GWAS: functional studies on the G6PC2 gene implicated in the regulation of fasting blood glucose. Curr Diab Rep. 2013;13(6):768-77. Epub 2013/10/22. doi: 10.1007/s11892-013-0422-8. PubMed PMID: 24142592; PubMed Central PMCID: PMCPmc4041587.

25. Kelliny C, Ekelund U, Andersen LB, Brage S, Loos RJ, Wareham NJ, et al. Common genetic determinants of glucose homeostasis in healthy children: the European Youth Heart Study. Diabetes. 2009;58(12):2939-45. Epub 2009/09/11. doi: 10.2337/db09-0374. PubMed PMID: 19741166; PubMed Central PMCID: PMCPmc2780884.

26. Sabatti C, Service SK, Hartikainen AL, Pouta A, Ripatti S, Brodsky J, et al. Genome-wide association analysis of metabolic traits in a birth cohort from a founder population. Nature genetics. 2009;41(1):35-46. Epub 2008/12/09. doi: 10.1038/ng.271. PubMed PMID: 19060910; PubMed Central PMCID: PMCPmc2687077.

27. Leal SM, Service SK, Teslovich TM, Fuchsberger C, Ramensky V, Yajnik P, et al. Re-sequencing Expands Our Understanding of the Phenotypic Impact of Variants at GWAS Loci. PLoS Genetics. 2014;10(1):e1004147. doi: 10.1371/journal.pgen.1004147.

28. Chen WM, Erdos MR, Jackson AU, Saxena R, Sanna S, Silver KD, et al. Variations in the G6PC2/ABCB11 genomic region are associated with fasting glucose levels. The Journal of clinical investigation. 2008;118(7):2620-8. Epub 2008/06/04. doi: 10.1172/jci34566. PubMed PMID: 18521185; PubMed Central PMCID: PMCPmc2398737.

29. Soranzo N, Sanna S, Wheeler E, Gieger C, Radke D, Dupuis J, et al. Common variants at 10 genomic loci influence hemoglobin A(1)(C) levels via glycemic and nonglycemic pathways. Diabetes. 2010;59(12):3229-39. Epub 2010/09/23. doi: 10.2337/db10-0502. PubMed PMID: 20858683; PubMed Central PMCID: PMCPmc2992787.

30. Ezzidi I, Mtiraoui N, Chaieb M, Kacem M, Mahjoub T, Almawi WY. Diabetic retinopathy, PAI-1 4G/5G and -844G/A polymorphisms, and changes in circulating PAI-1 levels in Tunisian type 2 diabetes patients. Diabetes Metab. 2009;35(3):214-9. doi: 10.1016/j.diabet.2008.12.002. PubMed PMID: 19419896.

31. Jiang G, Hu C, Tam CH, Lau ES, Wang Y, Luk AO, et al. Genetic and clinical variables identify predictors for chronic kidney disease in type 2 diabetes. Kidney Int. 2016;89(2):411-20. Epub 2016/01/26. doi: 10.1016/j.kint.2015.09.001. PubMed PMID: 26806836.
