# Supplementary figures and images for: Meta-analyses of the association of G6PC2 allele variants with elevated fasting glucose and type 2 diabetes
Source: PLoS One. 2017 Jul 13;12(7):e0181232. doi: 10.1371/journal.pone.0181232 (PMC5509327; doi:10.1371/journal.pone.0181232)

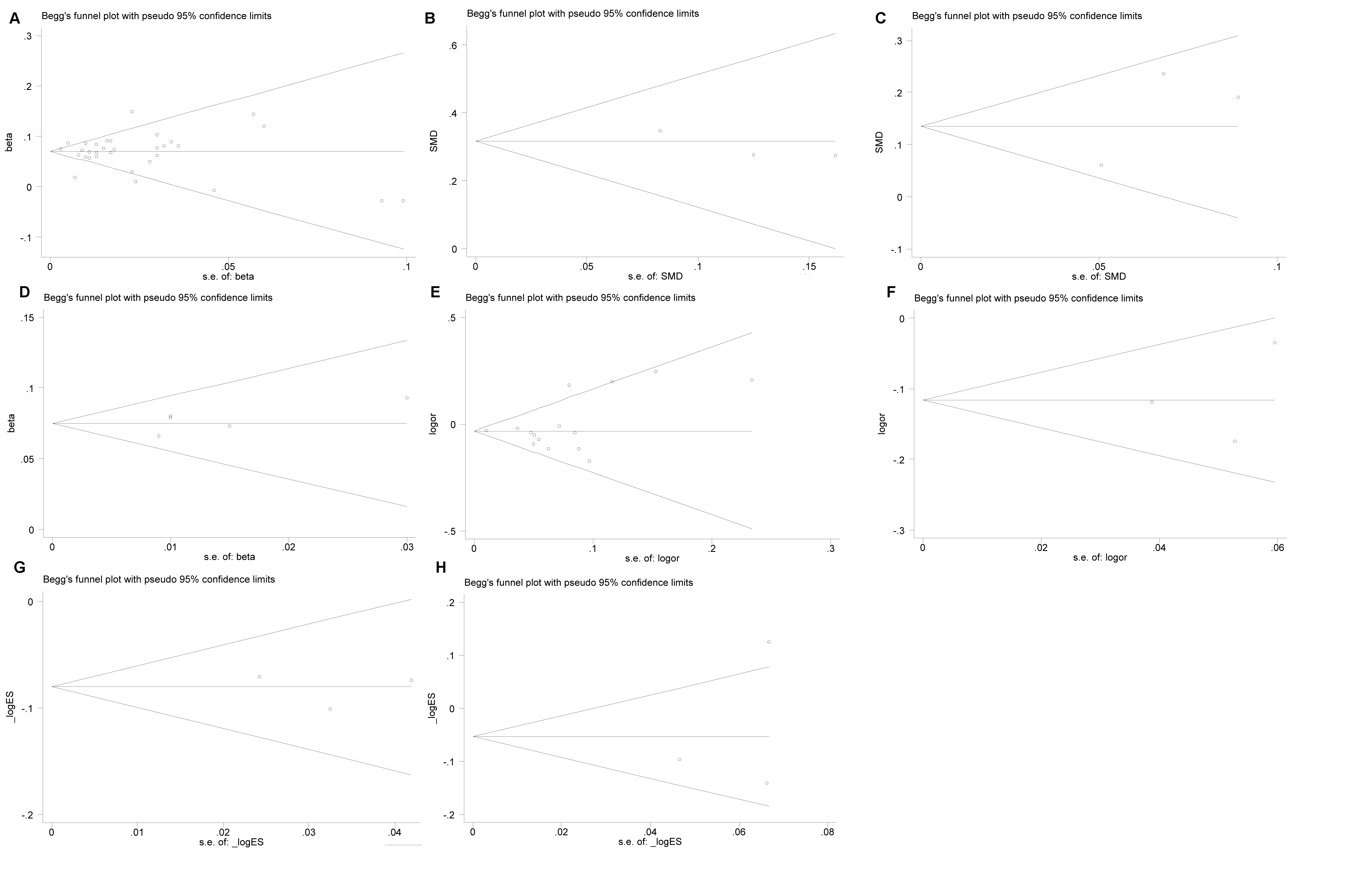

Supplement: S1 Fig — Funnel plot of publication bias for the association of rs560887 (A), rs16856187 (CCvsAA) (B) and (ACvsAA) (C), rs573225 (D) with FG, rs560887 (E), rs16856187 under allele (F), dominant (G) and recessive (H) with T2D, respectively. (TIF) [file pone.0181232.s005.tif]

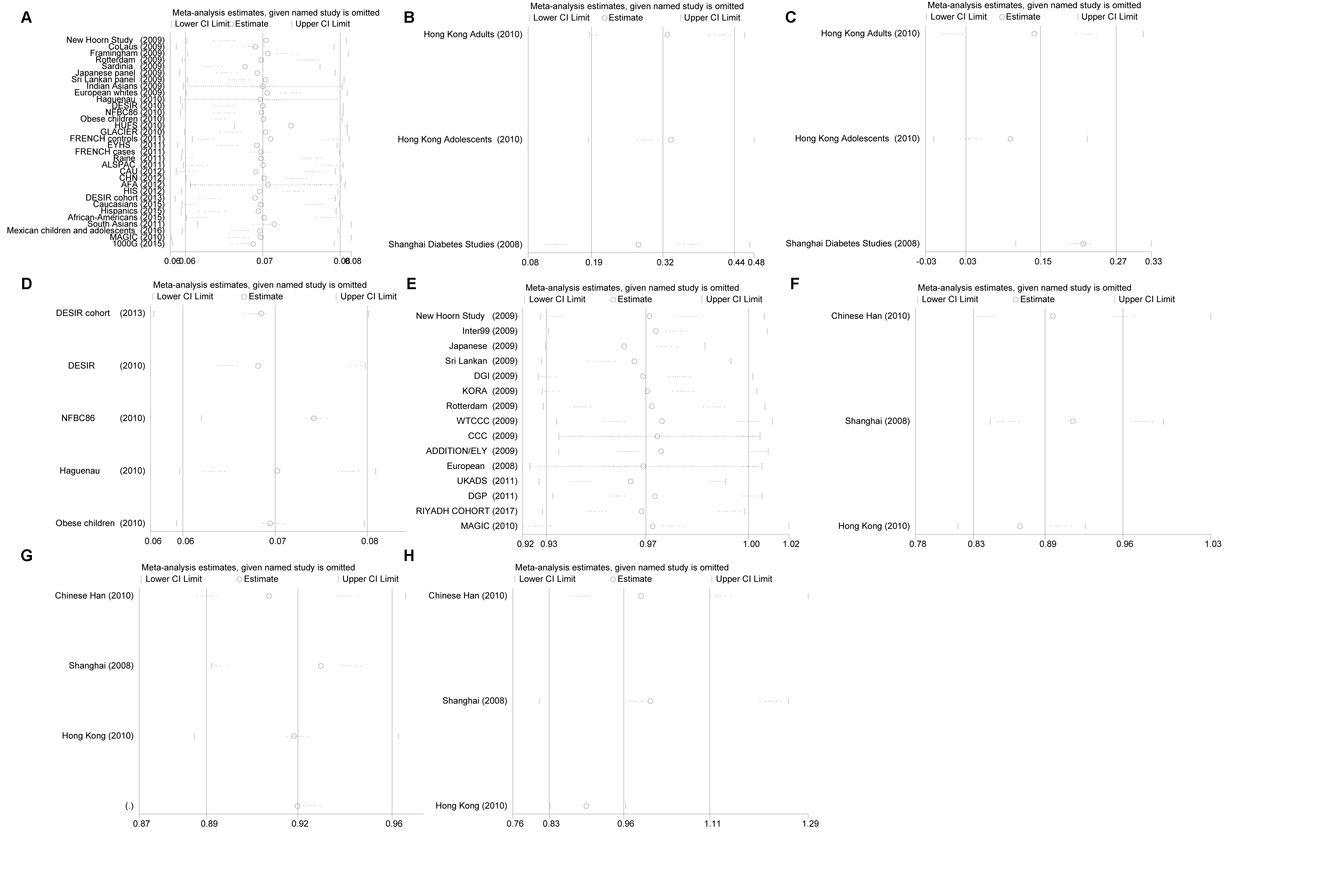

Supplement: S2 Fig — Sensitivity tests for the association of rs560887 (A), rs16856187 (CCvsAA) (B) and (ACvsAA) (C), rs573225 (D) with FG, rs560887 (E), rs16856187 under allele (F), dominant (G) and recessive (H) with T2D, respectively. (TIF) [file pone.0181232.s006.tif]
